# Supplementary material for: Oxone®-Mediated TEMPO-Oxidized Cellulose Nanomaterials form I and form II
Source: Molecules. 2020 Apr 17;25(8):1847. doi: 10.3390/molecules25081847 (PMC7221945; doi:10.3390/molecules25081847)
Supplement: Supplementary file 1 [file molecules-25-01847-s001.pdf]

molecules

Supplemental Table S1. XRD raw data used to determine crystallinity and crystallite size of OTO-CNM Form I, Form II, and MCC starting material including peak X position, peak intensity in counts (cts), peak area, and the full width at half maximum (FWHM) of the reported curve.

Plane	Sample	Peak X (2θ)	Peak Intensity (cts)	Peak Area (arb)	FWHM (2θ)
101	MCC	16.0	428	2024	4.43
	Form I	16.2	271	1335	4.62
	Form II	16.5	153	817	4.99
002	MCC	22.5	968	2957	2.87
	Form I	22.5	862	2353	2.57
	Form II	22.5	267	1711	5.99

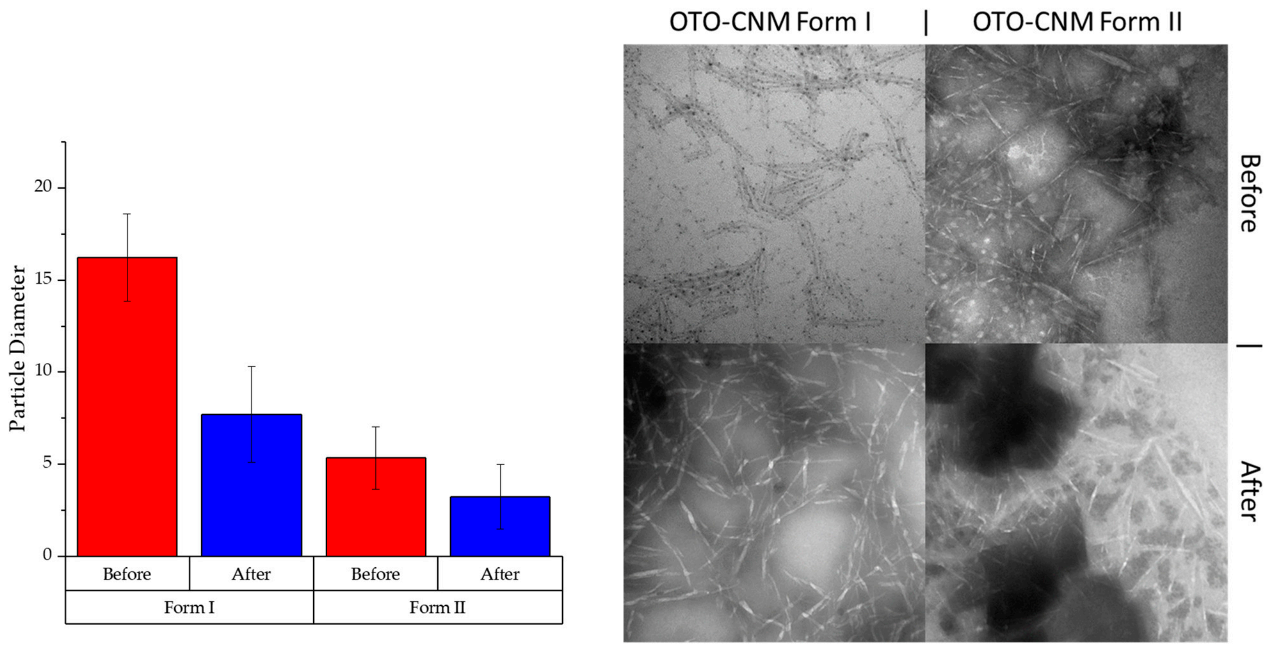

Supplemental Figure S1. TEM size analysis on OTO-CNM Form I and OTO-CNM Form II particle diameter before and after mechanical treatment. This figure shows the effect of mechanical treatment on the cellulose size after synthesis. While Form I is significantly affected by mechanical treatment post sonication, Form II show little change in size before and after mechanical treatment.
